# Supplementary material for: Autophagy impairment in liver CD11c+ cells promotes non-alcoholic fatty liver disease through production of IL-23
Source: Nat Commun. 2022 Mar 17;13:1440. doi: 10.1038/s41467-022-29174-y (PMC8931085; doi:10.1038/s41467-022-29174-y)

Autophagy impairment in liver CD11c$^+$ cells promotes non-alcoholic fatty liver disease through production of IL-23

Galle-Treger & Helou *et al.*, Supplementary information.

Supplementary Figure 1

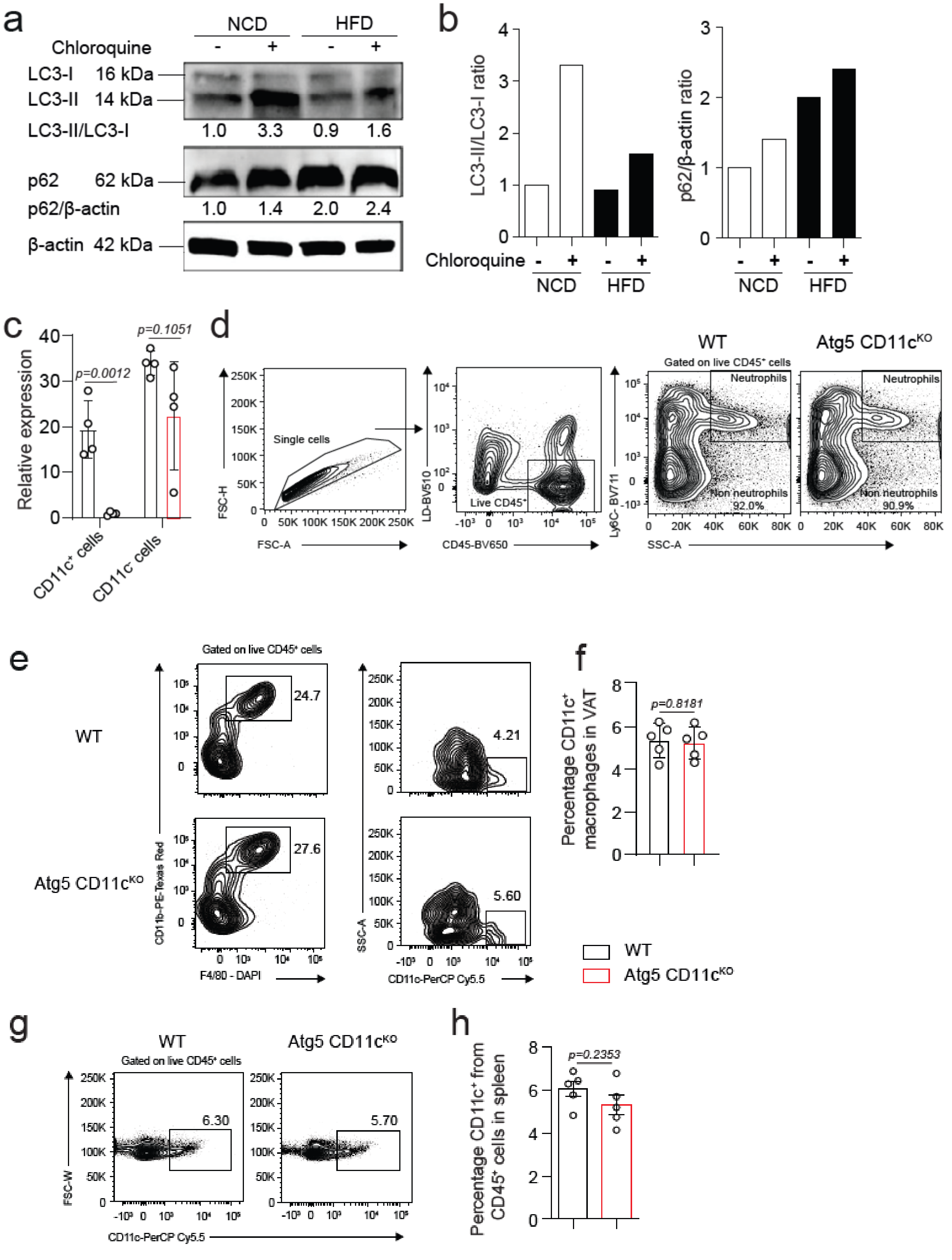

(a) Western blot analysis of LC3-II/LC3-I and p62 levels in CD11c$^+$ liver cells isolated from C57BL/6 mice fed normal chow diet (NCD) or high-fat diet (HFD) for 14 weeks and cultured for 15 hours in the presence or absence of chloroquine (25μM). **(b)** Densitometric quantification of the LC3-II/LC3-I and the p62/actin ratios. **(c)** Relative expression of Atg5 in CD11c$^+$ and CD11c$^-$ cells in liver from WT and Atg5 CD11c^{KO} mice, n=4 mice. **(d)** Exclusion of liver neutrophils identified as SSC^{high} Ly6C$^+$ CD45$^+$ cells. **(e)** Gating strategy of live CD11c$^+$ macrophages (CD11b$^+$ F4/80$^+$ CD45$^+$) in VAT from WT and Atg5 CD11c^{KO} mice fed NCD. **(f)** Percentage of CD11c$^+$ macrophages in VAT. **(g)** Representative dot plots of CD11c expression in splenocytes from WT and Atg5 CD11c^{KO} mice fed NCD. **(h)** Percentage of CD11c$^+$ splenocytes, n=5 mice. Error bars are the mean ± SD, two-tailed Student's t test.

<u>**Supplementary Figure 2**</u>

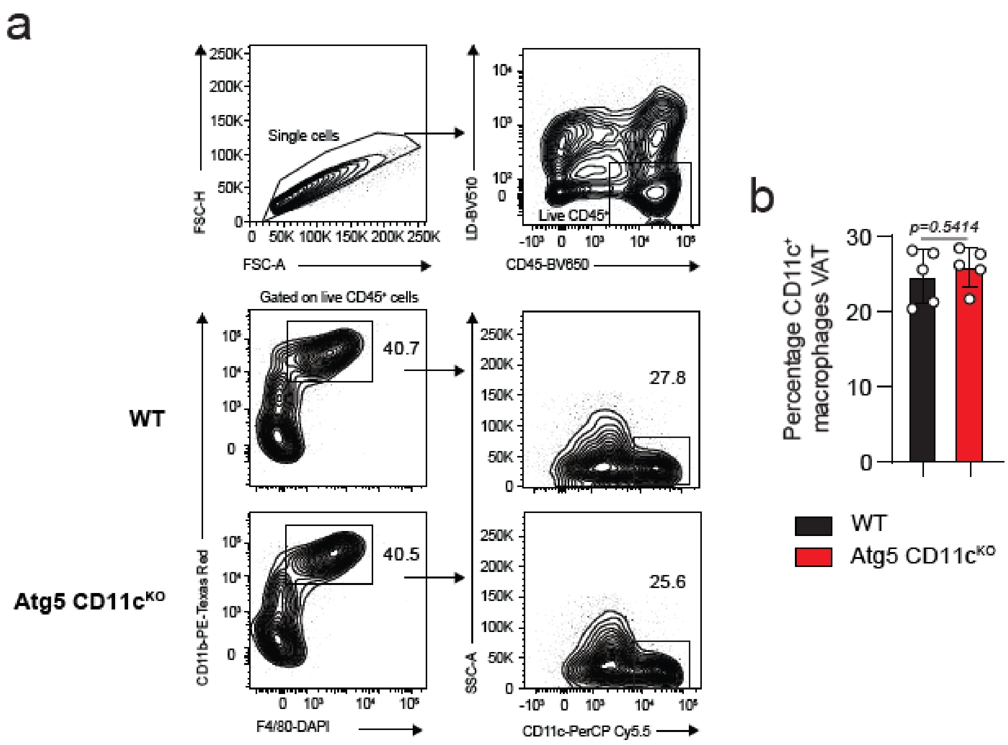

(a) Gating strategy of CD11c$^+$ macrophages (CD11b$^+$ F4/80$^+$ CD45$^+$) in VAT from WT and Atg5 CD11c^{KO} mice fed HFD for 14 weeks. **(b)** Percentage of CD11c$^+$ macrophages in VAT. Error bars are the mean ± SD, ns: non-significant, two-tailed Student's t test, n=5 mice. Experiments were performed three times.

Supplementary Figure 3

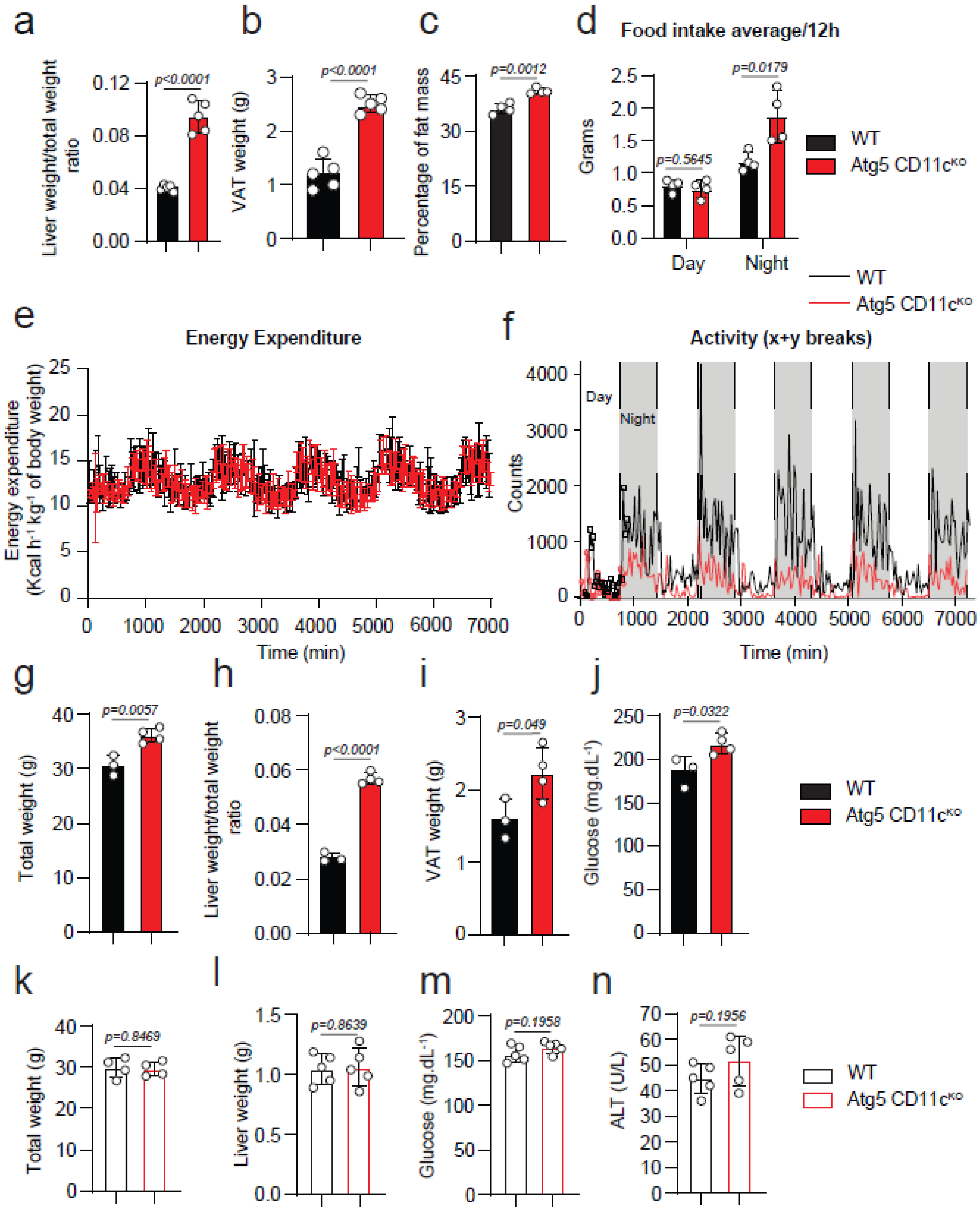

WT and Atg5 CD11c^{KO} mice were fed HFD for 14 weeks. **(a)** Ratios of liver weight: Total weight was calculated after 14 weeks of HFD. **(b)** VAT weight was measured after 14 weeks of HFD. **(c)** The percentage of fat mass was measured using a body composition analyzer. **(d)** Food intake

average/12h were measured. CLAMS analysis was performed using individually housed groups of WT and Atg5 CD11c^{KO} mice maintained on a HFD. **(e)** Energy expenditure normalized by total body weight was measured. **(f)** Physical activity was measured. WT and Atg5 CD11c^{KO} mice were cohoused after weaning and then fed HFD for 14 weeks. **(g)** Total weights, **(h)** liver weight/ total weight ratios, **(i)** VAT weights, and **(j)** fasting blood glucose were measured after 14 weeks of HFD. WT and Atg5 CD11c^{KO} mice were fed NCD for 14 weeks. **(k)** Total weights, **(l)** liver weights, **(m)** fasting blood glucose, and **(n)** ALT levels were measured after 14 weeks of NCD. Error bars are the mean ± SD, two-tailed Student's t test, n=3-5 mice. Experiments were performed three times.

Supplementary Figure 4

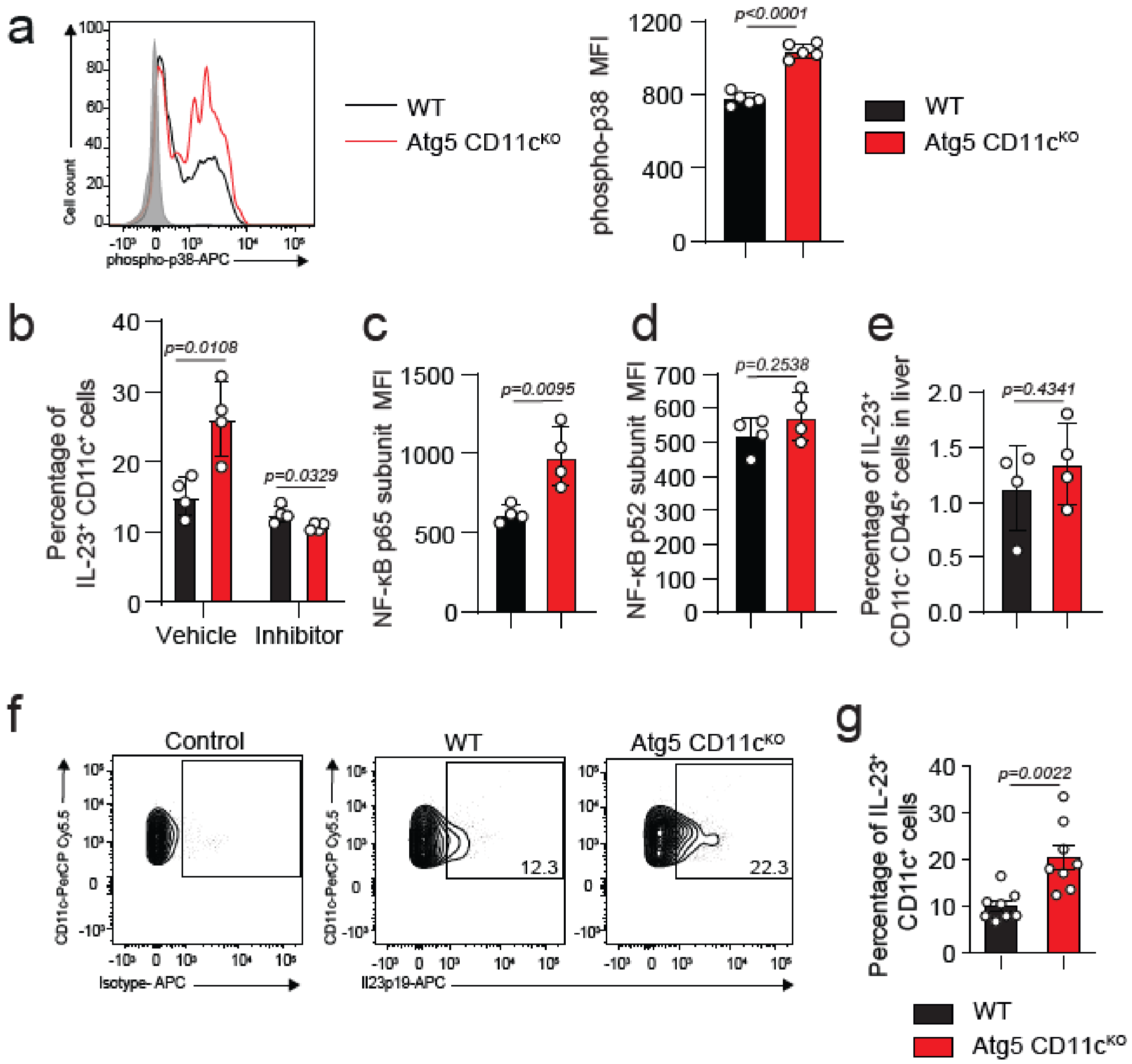

(a) Representative histogram of the expression of p38 in hepatic CD11c^{+} cells isolated from WT (black) and Atg5 CD11c^{KO} (red) mice fed HFD for 14 weeks. The level of isotype-matched stain control is shown as a grey-filled histogram (left). Corresponding quantification presented as Mean Fluorescence Intensity of p38 in hepatic WT and Atg5 CD11c^{KO} cells (right), n=5 mice. **(b)** Liver CD11c^{+} cells were isolated from WT and Atg5 CD11c^{KO} mice. Cells were stimulated ex-vivo in the presence of p38 inhibitor (10µM) or vehicle (DMSO) and IL-23 expression was assessed by flow cytometry. Percentage of IL23^{+} CD11c^{+} cells after 3 hours of incubation. **(c)** Active NF-κB p65

subunit and **(d)** NF-κB p52 subunit expressions were measured in hepatic $CD11c^+$ cells isolated from WT and Atg5 $CD11c^{KO}$ mice fed HFD for 14 weeks. **(e)** Percentage of $IL\text{-}23^+$ $CD11c^-$ $CD45^+$ cells in liver of WT and Atg5 $CD11c^{KO}$ mice fed HFD for 14 weeks, n=4 mice. **(f)** Representative flow cytometric plots of IL-23 expression in VAT $CD11c^+$ cells in WT and Atg5 $CD11c^{KO}$ mice after 14 weeks of HFD. **(g)** Percentage of VAT $IL\text{-}23^+$ $CD11c^+$ cells after 14 weeks of HFD, n=8 mice. Error bars are the mean ± SD, two-tailed Student's t test. Experiments were performed three times.

Supplementary Figure 5

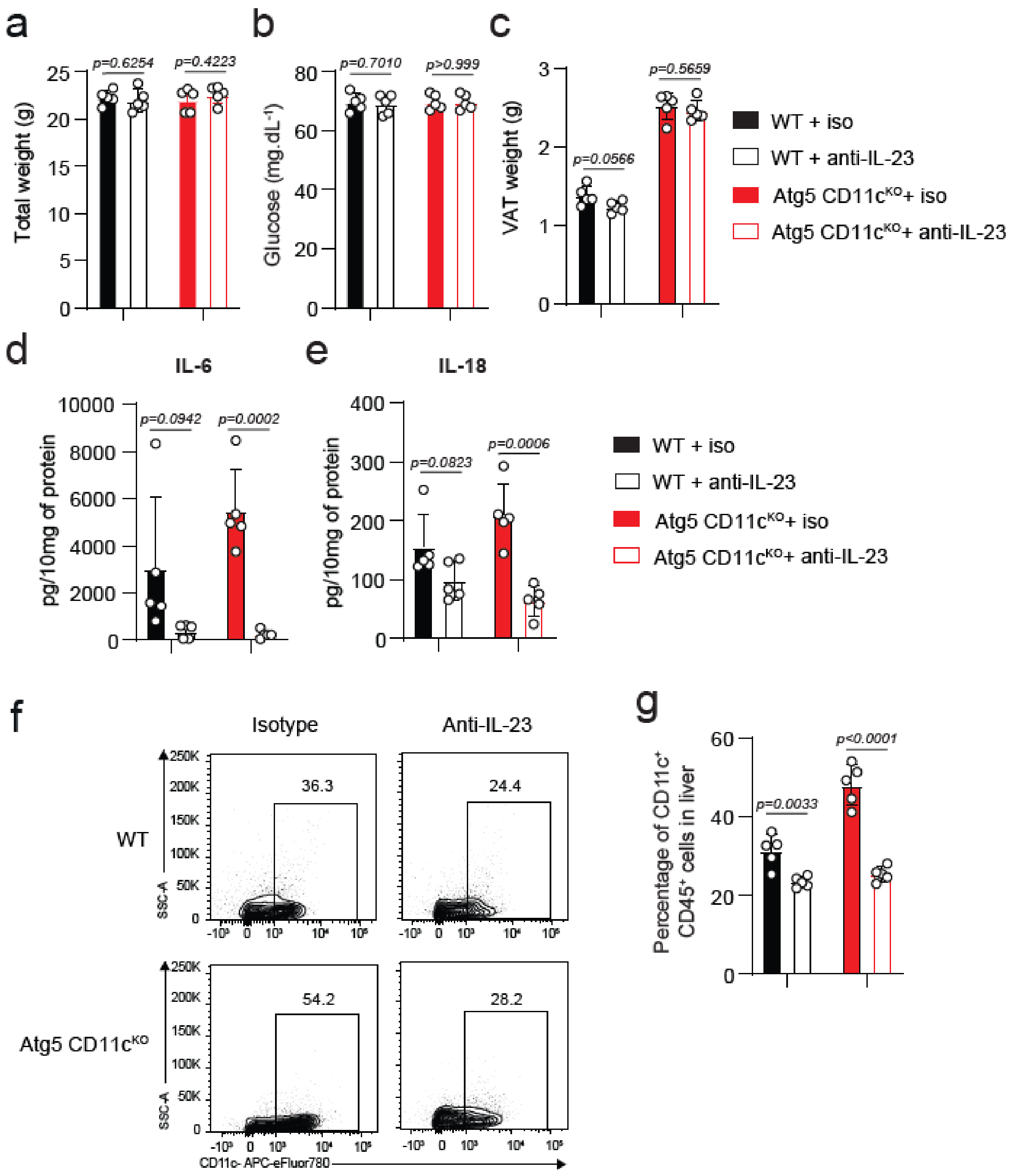

(a) Total weights and **(b)** blood glucose concentrations were measured in WT Atg5 CD11c^{KO} mice before anti-IL-23 treatment and HFD. **(c)** VAT weights were measured in WT and Atg5 CD11c^{KO} mice fed HFD for 14 weeks were treated either with anti-IL-23 antibody or isotype control. IL-6 **(d)**

and IL-18 **(e)** secretion levels were measured in liver lysates after 14 weeks of treatment. **(f)** Representative dot plots of CD11c expression in hepatic $CD45^+$ cells isolated from WT and Atg5 $CD11c^{KO}$ mice fed HFD for 14 weeks and treated either with anti-IL-23 antibody or isotype control. **(g)** Percentage of hepatic $CD11c^+$ $CD45^+$ cells after anti-IL-23 or isotype control treatment. Error bars are the mean ± SD, two-tailed Student's t test, n=5 mice. Experiments were performed three times.

Supplementary Figure 6

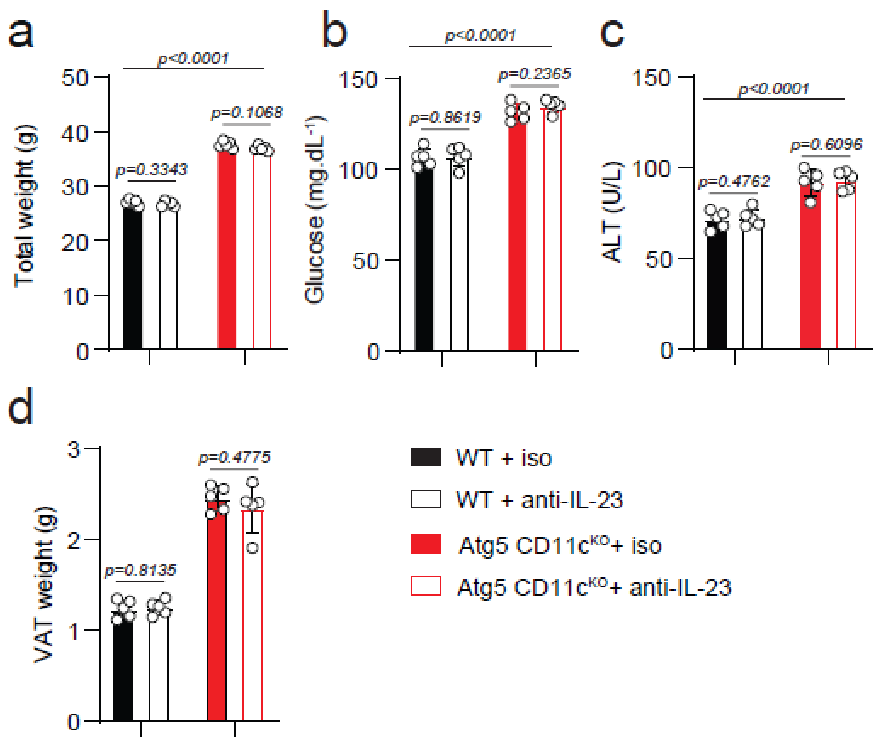

(a) Total weights, **(b)** blood glucose and **(c)** serum ALT levels were measured in WT and Atg5 CD11c^{KO} mice before anti-IL-23 treatment and after 8 weeks of HFD. **(d)** VAT weights were measured in WT and Atg5 CD11c^{KO} mice after anti-IL-23 or isotype control treatments which were started after 8 weeks of HFD. Error bars are the mean ± SD, two-tailed Student's t test, n=5 mice. Experiments were performed three times.

Uncropped scans of Supplementary Figure 1a blots

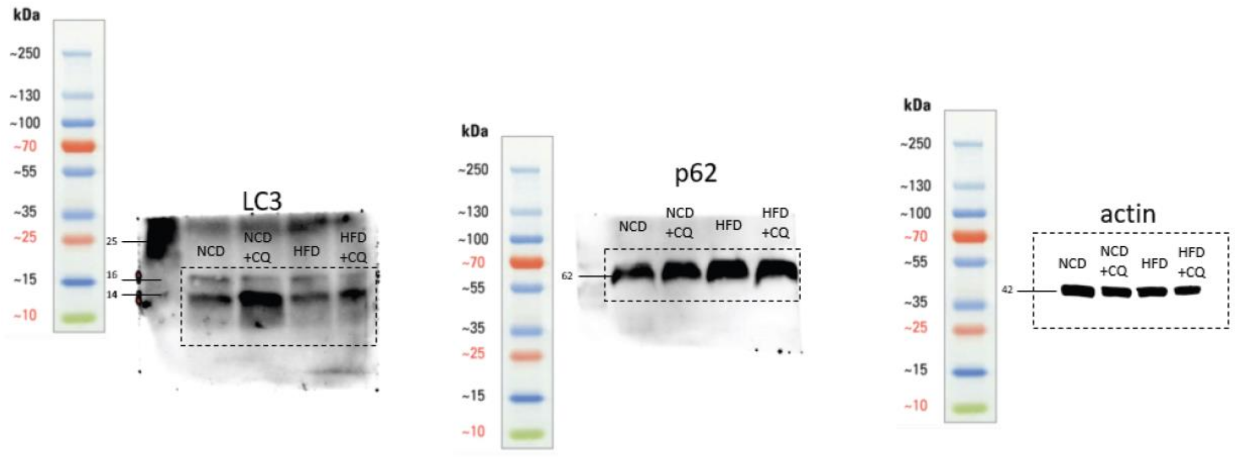

Supplement: Supplementary file 2 — Supplementary Information [file 41467_2022_29174_MOESM2_ESM.pdf]
