# Supplementary material for: Autophagy impairment in liver CD11c+ cells promotes non-alcoholic fatty liver disease through production of IL-23
Source: Nat Commun. 2022 Mar 17;13:1440. doi: 10.1038/s41467-022-29174-y (PMC8931085; doi:10.1038/s41467-022-29174-y)
Supplement: Supplementary file 3 — Reporting Summary [file 41467_2022_29174_MOESM3_ESM.pdf]

nature research

Corresponding author(s): Dr. Omid Akbari

Last updated by author(s): December 17, 2021

Reporting Summary

Nature Research wishes to improve the reproducibility of the work that we publish. This form provides structure for consistency and transparency in reporting. For further information on Nature Research policies, see our Editorial Policies and the Editorial Policy Checklist.

Statistics

For all statistical analyses, confirm that the following items are present in the figure legend, table legend, main text, or Methods section.

n/a	Confirmed	
☐	☒	The exact sample size (*n*) for each experimental group/condition, given as a discrete number and unit of measurement
☐	☒	A statement on whether measurements were taken from distinct samples or whether the same sample was measured repeatedly
☐	☒	The statistical test(s) used AND whether they are one- or two-sided<br>*Only common tests should be described solely by name; describe more complex techniques in the Methods section.*
☒	☐	A description of all covariates tested
☒	☐	A description of any assumptions or corrections, such as tests of normality and adjustment for multiple comparisons
☐	☒	A full description of the statistical parameters including central tendency (e.g. means) or other basic estimates (e.g. regression coefficient) AND variation (e.g. standard deviation) or associated estimates of uncertainty (e.g. confidence intervals)
☐	☒	For null hypothesis testing, the test statistic (e.g. *F*, *t*, *r*) with confidence intervals, effect sizes, degrees of freedom and *P* value noted<br>*Give P values as exact values whenever suitable.*
☒	☐	For Bayesian analysis, information on the choice of priors and Markov chain Monte Carlo settings
☒	☐	For hierarchical and complex designs, identification of the appropriate level for tests and full reporting of outcomes
☒	☐	Estimates of effect sizes (e.g. Cohen's *d*, Pearson's *r*), indicating how they were calculated

Our web collection on statistics for biologists contains articles on many of the points above.

Software and code

Policy information about availability of computer code

Data collection	BD FACSDiva software v8.0.1 was used for flow cytometry data aquisition. Histological sections were acquired using a Leica DME microscope and Leica ICC50HD camera.
Data analysis	Partek Genomics Suite software version 7.0 Copyright, Qiagen Ingenuity Pathway Analysis (IPA) software 2021, FlowJo software (TreeStar) version 10, Prism analysis software version 8, Leica LAS EZ software (LAS EZ 3.4).

For manuscripts utilizing custom algorithms or software that are central to the research but not yet described in published literature, software must be made available to editors and reviewers. We strongly encourage code deposition in a community repository (e.g. GitHub). See the Nature Research guidelines for submitting code & software for further information.

Data

Policy information about availability of data

All manuscripts must include a data availability statement. This statement should provide the following information, where applicable:

- Accession codes, unique identifiers, or web links for publicly available datasets
- A list of figures that have associated raw data
- A description of any restrictions on data availability

Source data are provided with this paper. The RNA-seq data generated in this study (WT vs Atg5 CD11cKO mice) have been deposited in the Genbank database under accession code GSE190819. Publicly available data were downloaded from Gene Expression Omnibus database under accession codes GSE49541 (40 mild NAFLD versus 32 advanced NAFLD patients17; DOI: 10.1053/j.gastro.2013.07.047) and GSE88818 (WT mice fed NCD or HFD for seven weeks20; DOI: 10.1038/srep40220).

Field-specific reporting

Please select the one below that is the best fit for your research. If you are not sure, read the appropriate sections before making your selection.

☒ Life sciences ☐ Behavioural & social sciences ☐ Ecological, evolutionary & environmental sciences

For a reference copy of the document with all sections, see nature.com/documents/nr-reporting-summary-flat.pdf

Life sciences study design

All studies must disclose on these points even when the disclosure is negative.

Sample size	We used ANOVA to calculate the sample size with the assumption of power of 80%, effect size of ~~50, and alpha less than 0.05.
Data exclusions	No animals were excluded. There was no anticipation of excluding animals once they underwent the experiments.
Replication	All attempts at replication were successful, experiments were repeated three times.
Randomization	Age-matched male mice were randomly allocated to experimental groups. Randomization process consisted of pooling all the mice in a large cage and random picking out and allocating to experimental groups. Publicly available clinical data were downloaded from Gene Expression Omnibus database under accession codes GSE49541 (40 mild NAFLD versus 32 advanced NAFLD patients17; DOI: 10.1053/j.gastro.2013.07.047).
Blinding	For RNA sequencing, metabolic colorimetric assays, Western blots, and histological experiments, investigators were blinded to group allocation during data collection. No blinding was done for flow cytometry experiments as the design and conditions prevent possible sources of bias. Blinding was not relevant in other experiments since the diet-induced phenotype in WT vs KO mice was visually noticeable.

Reporting for specific materials, systems and methods

We require information from authors about some types of materials, experimental systems and methods used in many studies. Here, indicate whether each material, system or method listed is relevant to your study. If you are not sure if a list item applies to your research, read the appropriate section before selecting a response.

Materials & experimental systems

n/a	Involved in the study	
☐	☒	Antibodies
☒	☐	Eukaryotic cell lines
☒	☐	Palaeontology and archaeology
☐	☒	Animals and other organisms
☒	☐	Human research participants
☒	☐	Clinical data
☒	☐	Dual use research of concern

Methods

n/a	Involved in the study	
☒	☐	ChIP-seq
☐	☒	Flow cytometry
☒	☐	MRI-based neuroimaging

Antibodies

Antibodies used

Antibodies purchased from eBioscience:
FITC anti-mouse CD45, clone 30-F11, cat#11-0451-85, lot 2041142
eFluor660 anti-mouse Il23p19, clone fc23cpg, cat# 50-7023-82, lot 4349114
APC anti-human/mouse P-p38, clone 4NIT4KK, cat# 17-9078-42, lot 4325050

Antibodies purchased from BioLegend:
APC-eFluor780 anti-mouse CD11c, clone N418, cat#117324, lot B263881
PerCP/Cy5.5 anti-mouse CD11c, clone N418, cat#117328, lot B256450
Alexa Fluor 647 anti-mouse Clec4f, clone 3E3F9, cat#156804, lot B308088
BV421 anti-mouse F4/80, clone BM8, cat#123132, lot B265669
PE-Cy7 anti-mouse Tim-4, clone RMT4-54, cat#130010, lot B317627
APC-Cy7 anti-mouse I-A/-E, clone M5/114.15.2, cat#107628, lot B306884
BV510 anti-mouse I-A/-E, clone M5/114.15.2, cat#107635, lot B244226
BV650 anti-mouse CD45 , clone 30-F11, cat#103151, lot B311212
PE anti-mouse CD64, clone X54-5/7.1, cat#139304, lot B323783
PE/Dazzle 594 anti-mouse/human CD11b, clone M1/70, cat#101256, lot B299254
BV711 anti-mouse Ly6C, clone HK1.4, cat#128037, lot B297640

Antibodies used for immunoblots:
LC3B Antibody (Cell Signaling Technology), Rabbit anti-mouse, cat#2775S, lot 13

SQSTM1/p62 Antibody (Cell Signaling Technology), Rabbit anti-mouse, cat#5114S, lot 6
Anti-β-Actin Antibody (C4) (Santa Cruz Biotechnology), mouse monoclonal IgG1 κ, cat#sc-47778, lot H1420
Goat anti Rabbit Secondary Antibody HRP (Invitrogen), cat#31460, lot VJ313046
Goat anti Mouse Secondary Antibody HRP (Invitrogen), cat#31430, lot VK316101

Validation

All antibodies are from commercial source and have been validated by the vendors and their validation data are available on the manufacturers' websites (BioLegend: https://www.biolegend.com/en-us/quality/product-development, eBioscience https://www.thermofisher.com/us/en/home/life-science/antibodies/ebioscience.html, Cell Signaling Technology and Santa Cruz Biotechnology). Manufacturers' datasheets provide quality testing methods, validated applications, reported applications, usage instructions, application notes, publications using the antibody and other relevant data.

Animals and other organisms

Policy information about studies involving animals; ARRIVE guidelines recommended for reporting animal research

Laboratory animals

Four- to eight-week-old mice males were used in the studies. Atg5flox/flox and LC3-GFP mice are a gift from Dr. Noboru Mizushima (Tokyo Medical and Dental University, Tokyo, Japan). Mice were screened by using PCR, and Atg5flox/flox homozygote CD11c-Cre homozygote mice were selected for the experiments. Atg5flox/flox mice were backcrossed to B6.Cg-Tg(Itgax-cre)1-1Reiz/J mice (CD11c-Cre, #008068, Jackson Laboratory) and bred in our facility. C57BL/6J (000664) mice were purchased from the Jackson laboratory and bred in our facility. Mice were maintained at macroenvironmental temperature of 21-22°C, humidity (48-52%), in a conventional 12:12 light/dark cycle with lights on at 6:00 a.m. and off at 6:00 p.m.

Wild animals

No wild animals were used in this study.

Field-collected samples

No field-collected samples were used in this study.

Ethics oversight

All the experiments described in this manuscript were approved IACUC of USC and in complete compliance with the guidelines of IACUC of USC. Animal facilities at the USC are AAALAC accredited.

Note that full information on the approval of the study protocol must also be provided in the manuscript.

Flow Cytometry

Plots

Confirm that:

☒ The axis labels state the marker and fluorochrome used (e.g. CD4-FITC).

☒ The axis scales are clearly visible. Include numbers along axes only for bottom left plot of group (a 'group' is an analysis of identical markers).

☒ All plots are contour plots with outliers or pseudocolor plots.

☒ A numerical value for number of cells or percentage (with statistics) is provided.

Methodology

Sample preparation

Livers were collected at the indicated times after transcardial perfusion to clear organs of red blood cells. Livers samples were digested in collagenase IV (MP Biomedicals, LLC) at 37°C for one hour and then processed on a 70µm nylon cell strainer (Falcon®) into a single cell suspension. Liver samples were resuspended in 30% Percoll solution and centrifuged at 750g for 20min without brake. Supernatants were discarded and pellets were resuspended in RBC Lysis Buffer (Biolegend) for 5 min. Cells were then washed and ready for staining.

Instrument

Stained cells were analyzed on FACSCanto II and/or FACSARIA III systems (Becton Dickinson)

Software

The software used to collect samples is BD Facsdiva and the data were analyzed with FlowJo version 10 software (TreeStar, Ashland, Oregon)

Cell population abundance

50 000 live CD45+ CD11c+ liver myeloid cells were sorted per mouse. Purity was assessed by analyzing sorted cells for the same markers used for sorting. Purity greater than or equal to 90% was considered satisfactory.

Gating strategy

Relevant gating strategies of CD111c+ cells in liver, spleen and visceral adipose tissue are shown in Figure 2(a,c), Figure 3a, and Supp Figure 1 (d, e, g) and Supp Fig 2a, Supp Fig 4 (a,f) and Supp Fig 5f. Briefly, the first step consisted of debris exclusion and the selection of cells on a FSC-A/SSC-A scale based on known size and granularity. Doublets that deviated from a linear increase were excluded on a FSC-A/FSC-H scale. CD11c+ cells were gated from live CD45+ cells as shown in Figure 2a. The full gating strategy of macrophages and dendritic cells was provided in Figure 3a. The boundaries between negative and positive populations were defined according to isotype and full minus one (FMO) controls

☒ Tick this box to confirm that a figure exemplifying the gating strategy is provided in the Supplementary Information.
